# Do Basal Ganglia Amplify Willed Action by Stochastic Resonance? A Model

**DOI:** 10.1371/journal.pone.0075657

**Published:** 2013-11-26

**Authors:** V. Srinivasa Chakravarthy

**Affiliations:** Department of Biotechnology, Indian Institute of Technology, Madras, Chennai, India; University of Adelaide, Australia

## Abstract

Basal ganglia are usually attributed a role in facilitating willed action, which is found to be impaired in Parkinson's disease, a pathology of basal ganglia. We hypothesize that basal ganglia possess the machinery to amplify will signals, presumably weak, by stochastic resonance. Recently we proposed a computational model of Parkinsonian reaching, in which the contributions from basal ganglia aid the motor cortex in learning to reach. The model was cast in reinforcement learning framework. We now show that the above basal ganglia computational model has all the ingredients of stochastic resonance process. In the proposed computational model, we consider the problem of moving an arm from a rest position to a target position: the two positions correspond to two extrema of the value function. A single kick (a half-wave of sinusoid, of sufficiently low amplitude) given to the system in resting position, succeeds in taking the system to the target position, with high probability, only at a critical noise level. But for suboptimal noise levels, the model arm's movements resemble Parkinsonian movement symptoms like akinetic rigidity (low noise) and dyskinesias (high noise).

## Introduction

Willed actions are a form of voluntary actions, though no rigorous definition of willed action is available [1]. Voluntary actions are characterized by presence of a goal, a plan to achieve that goal, conscious awareness of the action being performed, and an intention behind the whole process. William James [2] offers a further classification of voluntary actions into ideo-motor actions and willed actions. In the former, a pre-existent idea of how the action has to be performed is simply executed. On the contrary, in willed action, there is no pre-existent idea but only the pure, direct action of will driving and shaping movement. In James' own words, in case of willed actions, in contrast to ideo-motor actions, there is “an additional conscious element in the shape of a fiat, a mandate, or expressed consent” [2].

Willed actions are also defined in terms of internal vs. external sources of movement control. Accordingly, willed actions are those that are not triggered by external stimuli and are generated by internal sources [3], though there could be an admixture of external, sensory information once the ball is set in motion.

The work by Kornhuber and Deecke [4], [5] may be described as one of the earliest instances of a search for neural substrates of willed action. Analysis of Electroencephalogram (EEG) data from normal subjects engaged in self-initiated wrist movement showed a special potential that builds up over the midline central electrode (*C*_z_) more than a second before the movement begins. This activity, termed the Bereitschaftspotential (BP), or the Readiness Potential (RP), is found to be maximal at the midline centro-parietal area, and to be distributed bilaterally regardless of the site of movement. The activity, however, becomes localized to the contralateral side of the movement, as the movement onset time draws near. Dipole analysis of the sources of BP throws up the Supplementary Motor Area (SMA) as a key area responsible for BP [6].

Subsequent work on cortical substrates of willed action revealed other cortical sites also. Positron Emission Tomography-based studies on substrates for random finger lifting revealed marked activation of dorsolateral prefrontal cortex and anterior cingulate cortex [7]. In another study in which the subjects made random movements of a joy-stick in one of possible four directions (forward, backward, left and right) the cortical areas that showed preferential activation were, in addition to SMA, dorsolateral prefrontal cortex, anterior cingulate cortex and also premotor cortex [8].

Other subcortical structures, most importantly the frontostriatal circuits, were also found to be involved in willed action [1]. The frontostriatal circuits form loops that arise from frontal areas and run through the basal ganglia (BG) nuclei [9]. These loops are also thought to be organized into well-segregated multiple sub loops named as – the skeletomotor, oculomotor, associative/prefrontal and limbic loops [10]. It is tempting to assume that each of the sub loops is dedicated to a certain aspect of voluntary action. The idea that BG circuits are reasonably well-segregated into parallel sub loops is an old one and emerges out of earliest studies by Alexander et al [10], Albin et al [9] and some recent studies too [11]. There were also studies that reveal a functional segregation of BG circuits into subloops [12]–[15]. However, it must be immediately pointed out that the existence of segregated functional sub loops is not critical for the validity of the proposed model, as long as it is granted that the BG circuit as a whole can contribute to reaching through the instrumentality of its reinforcement learning machinery.

Damage to specific modules in the frontostriatal system is known to cause specific impairments in motor and cognitive functions. Prefrontal lesions are linked to perseveration, distractibility, impaired planning of sequential movements, and a tendency to shift from self-initiated behavior to stimulus-driven behavior [16]. Damage to SMA is associated with diminished spontaneous movements and partial mutism. Impairment in performing complex simultaneous or sequential movements is seen relative to performance of simple movements in a patient with right SMA damage [17].

The neuromodulator, dopamine, is known to play a crucial role in the function and coordination of frontostriatal circuits [18]. Dopamine cells in the Substantia Nigra pars compacta (SNc) project extensively to the striatum and other BG nuclei. The idea that activity of mesencephalic dopaminergic cells represents some sort of reward to the organism provides important clues to our understanding of BG [19]. Since the striatum also receives extensive afferents from sensory-motor cortex, it places BG in a unique position for selecting rewarding actions among a host of competing actions. Thus BG may be viewed as the neural machinery necessary for performing reinforcement learning (RL), a type of learning in which stimulus-response associations that maximize reward are reinforced. A vast body of modeling effort is driven by application of RL concepts to BG function [20]–[22]. Reinforcement Learning-based models are able to explain a wide array of phenomena related to BG function and fronto-striatal interactions in normal function and disease [23]–[25].

In Parkinson's disease (PD), a neurodegenerative disorder associated with loss of dopamine cells in SNc, motor symptoms like akinesia or bradykinesia and tremor are observed [26]. Parkinson's disease patients also exhibit difficulty in movement initiation. A more dramatic case of movement initiation exhibited by PD patients is the phenomenon of freezing of gait, which refers to difficulty in proceeding with gait [27]. This can happen in the beginning of a walk (start hesitation) or when trying to make a sharp turn (turning hesitation) [27]. Parkinson's disease patients also exhibit articulatory freezing, a kind of difficulty in speech initiation [28]. The aforementioned symptoms are negative symptoms of PD, marked by paucity of movement, often seen under conditions of OFF medication. On the other hand, under conditions of ON medication, PD patients exhibit uncontrolled, exaggerated movements like dyskinesias and chorea-like movements [26].

In the present study, we describe a model of willed action with BG as a key substrate. The model presents the conditions for normal willed action and its impairment under conditions of damaged BG. We assume that the “will” signal is weak, compared to the “bottom-up” signals derived from the sensory stream, and therefore needs appropriate machinery for amplification. We propose that by affording a combination of gradient descent and noise, BG serves as an excellent substrate for SR phenomenon, and amplifies the weak willed action signal arising from the prefrontal cortex or SMA. Stochastic resonance is a counter-intuitive effect by which the signal-to-noise ratio (SNR) of a nonlinear system or a device is highest when a moderate level of noise is added to the system; SNR is lower for both higher and lower noise levels [29]. Similarly in the proposed model of willed action, highest amplification is obtained at optimal noise level, which corresponds to normal function. Deviations from this optimal noise level are manifest as failure to initiate movement (low noise case) or unregulated movement (high noise case), reminiscent of motor symptoms of PD patients.

The paper is organized as follows: Section 2.1 summarizes a reaching model involving BG and motor cortex. The relation between the reaching model and SR dynamics is elucidated in section on Methods. The conditions under which maximal amplification of the will signal is achieved, is explored numerically in the subsequent section. Effects of deviations from the optimal noise level are also described in the same section. A discussion of simulation results, a more detailed neurobiological interpretation of the proposed model, and model limitations are presented in the subsequent sections. Conclusions of the study are presented in the final section.

## Methods

### 2.1 Background

The starting point of the present work is a model of reaching that highlights the role of BG [24]. This model is built on the general understanding that BG are essential for motor learning [30]. Cast in RL framework, the model depicts how BG enables the motor cortex to learn to reach a target location on command. The model consists of three components: motor cortex (MC), BG, and the arm. The arm has to reach one of 4 target locations. Each target is specified by a Target Selection Vector (TSV), *ξ*, which is given as input to the MC. In response to TSV, the MC produces muscle activation vector, *g*_m_. The BG component also outputs a muscle activation vector, *g*_bg_, which is combined with that of MC, to produce a final muscle activation vector, *g*, given as:
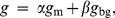
(2.1.1)where *α* and *β* are coefficients that control the relative contributions of MC and BG to movement. In eqn. (2.1.1), *g* denotes the neural activations given to the muscles of the arm. A given *g* places the arm in a unique configuration.

#### Arm model

Since BG dynamics is the focus of the paper, we chose an extremely simple model of arm dynamics. The arm consists of two joints with 4 muscles. The muscles are activated by *g*, a 4-dimensional vector: *g*_1_ and *g*_2_ activate the agonist and antagonist of the shoulder respectively, while *g*_3_ and *g*_4_ activate the agonist and antagonist of the forearm respectively. The shoulder and forearm joint angles, *θ*_1_ and *θ*_2_, respectively, are given by:
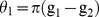
(2.1.2a)

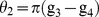
(2.1.2b)where 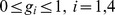.

Thus in our simple arm model, the relationship between muscle activations and arm configuration is a static one.

We now outline how BG enables MC to learn to reach a target, by producing muscle activations, *g*_m_, appropriate for a given TSV. In the early stages of learning, since MC is in untrained condition, *g*_m_ is expected to be off the mark. But the BG output *g*_bg_, which also represents muscle activations, is a highly labile quantity which perturbs *g*_m_ until the arm makes a successful reach. The value of *g*_bg_ which results in a successful reach is used by MC for training itself. Neurobiological interpretation of *g*_m_ and *g*_bg_ needs a comment. That the MC encodes muscle activations is a familiar idea [31]. But there is also evidence that neurons in putamen code for muscle activation patterns in addition to kinematic information [32].

As mentioned above, *α* and *β* control the relative contributions of MC and BG to the arm. In the early stages of learning, *α* is small, and movement is determined predominantly by BG output, whereas in late stages, MC dominates movement. (*β* is small; refer to [24] for more details)

#### Motor Cortex (MC)

The motor cortex is modeled as a perceptron with *ξ* as input and *g*_m_ as output.
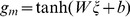
(2.1.3)


#### Basal Ganglia Model

The BG circuit receives inputs from the cortex and sends projections back to the cortex via the thalamus. The striatum is the input port of the BG, while the key output ports are Substantia Nigra pars reticulata (SNr) and the Globus Pallidus interna (GPi). The striatum projects directly to the output ports over the so-called Direct Pathway (DP) and indirectly over the Indirect Pathway (IP) with two intermediate stages – Globus Pallidus externa (GPe) and the Subthalamic nucleus (STN). Dopaminergic cells of Substantia Nigra pars compacta (SNc) project to striatum and other targets in BG. Therefore, the BG part of the model has 4 key components – 1) the Critic representing the Striatum [33], 2) the Direct Pathway (DP), 3) the Indirect Pathway (IP) and 4) the Temporal Difference (TD) error, *δ*, representing the SNc DA signal. The Critic assesses the current position of the arm's end effector with respect to the target. The DP and IP of BG take the change in the BG output, Δ*g*_bg_(*t*), in the current step, and update it to Δ*g*_bg_ (*t*+1). TD error, *δ*, is used to calculate Δ*g*_bg_ (*t*+1) using Δ*g*_bg_(*t*).

These components are defined below.

Critic: The Critic computes the Value function, which is a function of the distance, *d*, between the arm's end effector and the target.
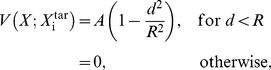
(2.1.4)where,
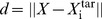


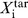
 = target position, *X* = current position, *V* is Value function. The norm (∥•∥) used is Euclidean norm.

**Figure 1:**
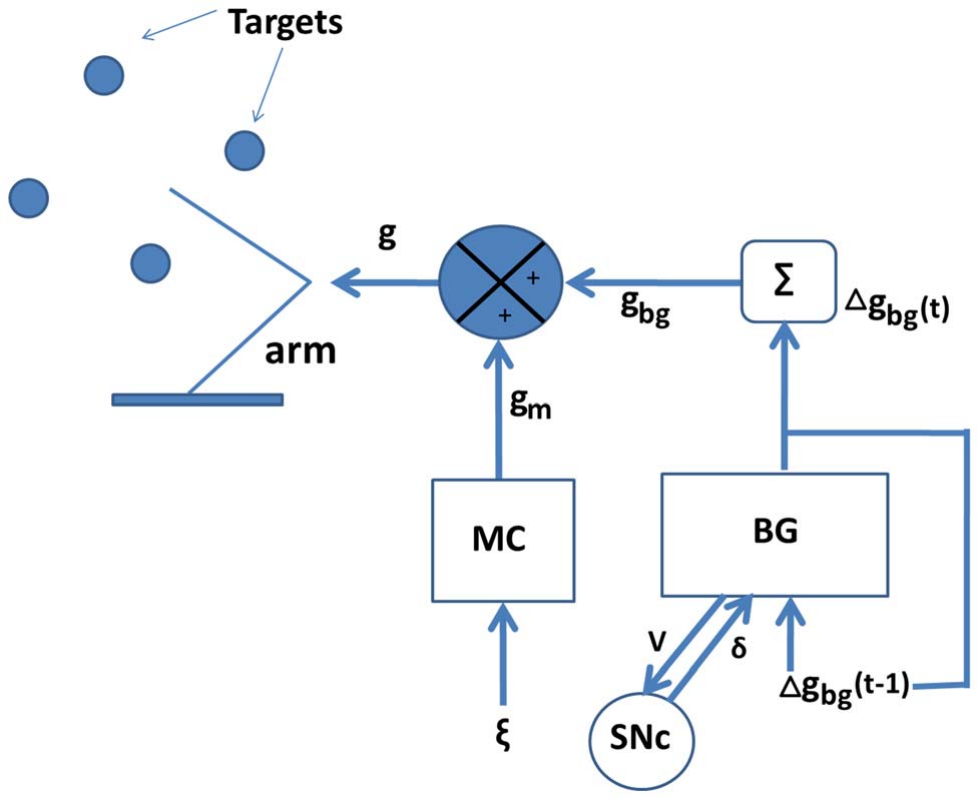
A model of reaching involving basal ganglia (redrawn based on [24]).

Dopamine signal: The dopamine signal represents TD error, *δ*.
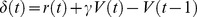
(2.1.5)The reward, *r*(*t*) = *A*, when *d* < *R*_small_ , otherwise *r*(*t*) = 0, where *R*_small_ is a small positive quantity. *δ* is thought to be computed within the loop: Striatum → SNc → Striatum (fig. 1). We let *γ* = 1, in the present model. Since *r*(*t*) is non-zero only when the target is reached, all along the trajectory *δ*(*t*) simply represents the temporal difference Δ*V* = *V*(*t*) – *V*(*t*−1).

Direct and Indirect Pathways (DP & IP): The next value of BG output, Δ*g*_bg_(*t*+1), is computed in the DP and IP of BG, as a function of *δ*(*t*) and Δ*g*_bg_(*t*) as follows:
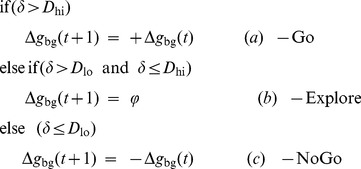
(2.1.6)where *φ* is a random four-dimensional vector such that norm (*φ*) = *η*, where norm(•) refers to Euclidean norm and *η* is a constant. Here *g*_bg_ is updated such that *g*_bg_ (*t*+1) = *g*_bg_(*t*) + Δ*g*_bg_(*t*). Adding the term *κ*Δ*g*_bg_(*t*−1), where 0<*κ*<1, to the Right Hand Side (RHS) of eqns.(2.1.6,abc) has a stabilizing effect on the arm's movements. Here *D*_lo_ and *D*_hi_ are thresholds that define the regimes.

Training MC: Learning occurs only in the MC. The dynamics of eqn. (2.1.6) proceeds until the end-effector comes close to the target location (*r*<*R*_tol_). The value of *g*, which results in this successful reach, is used as target output of MC, which is trained by delta rule as follows:
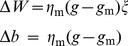
(2.1.7)where *η*_m_ is the learning rate.

Let us revisit eqns. (2.1.6a,b,c) which are crucial in making the connection between the above reaching model and the proposed SR dynamics subserved by the BG. In eqns. (2.1.6a,b,c), a positive *δ* represents approach towards the target, while a negative *δ* represents withdrawal away from the target. If a given Δ*g*_bg_ produces a sufficiently large positive *δ*, and hence a significant excursion towards the target, in one step, it is desirable to move in the same direction in the next step; therefore Δ*g*_bg_(*t*) = Δ*g*_bg_(*t*−1) in eqn. (2.1.6a). If a given Δ*g*_bg_ produces a sufficiently large negative *δ*, and hence a significant excursion away from the target, in one step, it is desirable to move in the opposite direction in the next step; therefore Δ*g*_bg_(*t*) = − Δ*g*_bg_(*t*−1) in eqn. (2.1.6c). If *δ* is small in magnitude, the previous movement is neutral, neither significantly towards or away from the target; therefore new directions are explored in the next step (Δ*g*_bg_(*t*) is random in eqn. (2.1.6b)). Such BG dynamics implies an expansion of classical Go-NoGo depiction of BG function [34].

**Figure 2:**
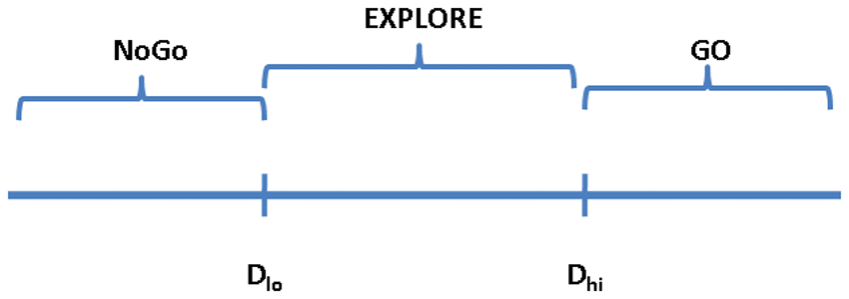
The Go, Explore and NoGo regimes of BG.

According to classical functional depictions of BG, striatal dopamine switches the transmission between DP and IP: the DP is selected at higher values of dopamine, while the IP for lower values [35]. Selection of DP is thought to facilitate movement (Go) and selection of IP to withhold movement (NoGo). Between the high and low ranges of dopamine, which correspond to the classical Go and NoGo regimes, we posit an intermediate range, which corresponds to Explore regime (fig. 2) [34]. These three regimes operate in the current model as follows. In the Go case, the DP is activated and *g*_bg_ is updated such that the arm continues to move a little in the previous direction. In the NoGo case, the IP is activated and *g*_bg_ is updated such that the arm shows a tendency to move a little in the direction opposite to the previous direction. In the explore case, again the IP is activated and *g*_bg_ is updated in a random fashion unrelated to the previous increment in *g*. Using a network model of BG, we have recently shown how, in addition to the Go and NoGo regimes, the new explore regime emerges naturally out of complex dynamics of the Subthalamic Nucleus (STN) – Globus Pallidus externa (GPe) loop in BG [34].

The dynamics of reaching described above has essentially two components: hill-climbing over value function, *V*(*t*), and a stochastic component that corresponds to exploration. Alternatively, this can be seen as a combination of gradient-descent over a new potential function defined as, *V*_p_ = −*V*, and stochastic dynamics. This combination of gradient descent and stochastic dynamics is the typical recipe for SR [29]. We have shown in Appendix that the BG eqns. (2.1.6a,b,c) closely resemble SR dynamics. We pursue the consequences of this analogy now.

### 2.2 Stochastic Resonance and Basal Ganglia Dynamics

A simple, standard version of a SR system can be expressed as [29]:
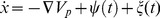
(2.2.1)


The first term on RHS is the gradient of a potential function *V*_p_ (*x*); the second term, *ψ*(*t*), represents noise; the third term *ξ*(*t*), denotes a weak signal that must be amplified by SR dynamics.

As shown in Text S1, eqn. (2.1.6) that describes BG dynamics, can be expressed as a differential equation that closely resembles the SR dynamics of eqn. (2.2.1) as follows,
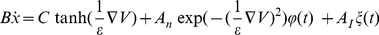
(2.2.2)


The first term on the RHS of eqn. (2.2.2) represents both Go (eqn. 2.1.6a) and NoGo (eqn. 2.1.6c) dynamics of BG. The second term represents the exploratory dynamics of eqn. (2.1.6b). The third term *ξ*(*t*), denotes the will signal, arising from cortex. Neural substrates of the will signal are discussed in the Discussion section.

**Figure 3:**
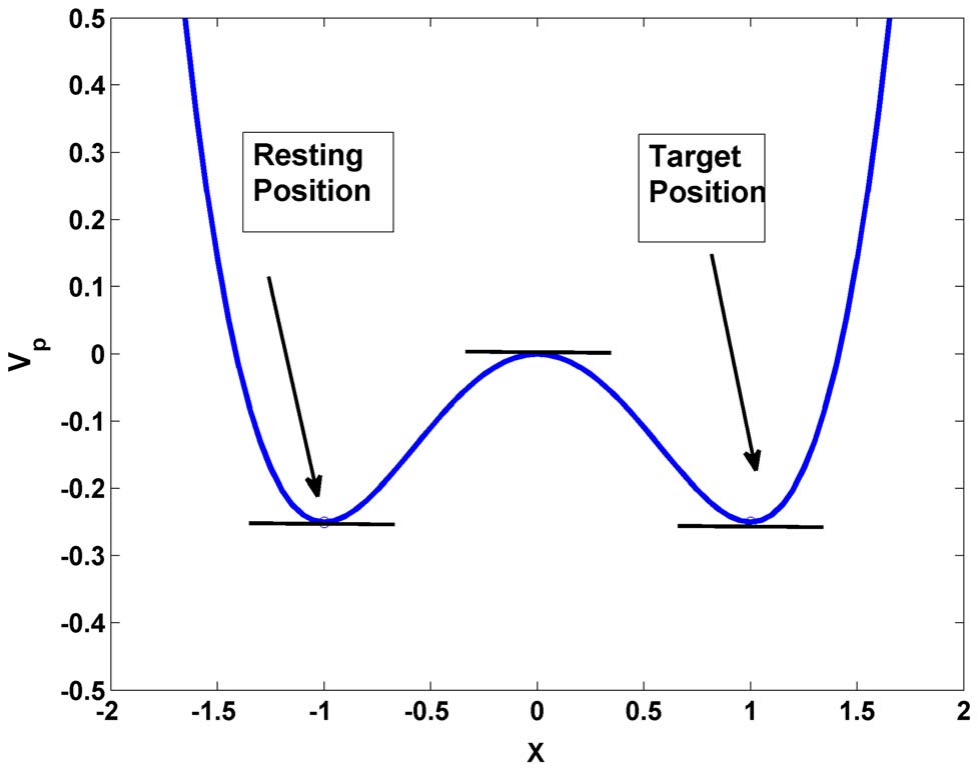
Bistable potential defined by eqn. (2.2.3). The resting position corresponds to (*x* = −1, *V*_p_ = −0.25) and the target position to (*x* = 1, *V*_p_ = −0.25).

Let us assume that *V*_p_(*x*) is a bistable potential well (fig. 3), given by:
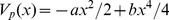
(2.2.3)where the two minima of *V*_p_(*x*) denote two stable states of the arm: 1) the resting position at *x* = −1, and 2) the target position at *x* = 1. Note that *V* in eqn. (2.2.2) is related to *V*_p_ in eqn. (2.2.3) as, *V*_p_ = −*V*.

Reaching is achieved by switching the system from the resting position (*x* = −1) to the target position (*x* = 1). This switching is done by presenting appropriate *ξ* (*t*), which delivers a kick in the form of a half-wave sinusoid:
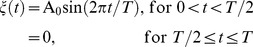
(2.2.4)


We also assume that noise is injected only during the time the kick (eqn. 2.2.4) is delivered. Thus the noise model is:
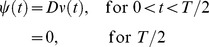
(2.2.5)where *δ* is a Gaussian random variable (mean = 0, SD = 1).

We now study the conditions under which the system makes a successful reach, by transitioning from the resting position to target position.

## Results

The idea explored in this study is the possibility that BG amplify will signal, presumably weak, by SR. That is, the will signal *ξ*(*t*), is incapable of producing a reach by itself. But when aided by the noise arising from BG, this originally subthreshold signal crosses a threshold and results in movement. Assuming *ξ*(*t*) to be a constant, *ξ*_0_, let us consider the minimum value of *ξ*_0_ necessary to make a transition from the resting position to the target position, under noise-free conditions. For the potential of eqn. (2.2.3), the dynamics is expressed as:
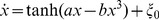
(3.1)For *ξ*_0_ = 0, eqn. (3.1) has three equilibrium points, two of them stable (*x* = ± (*a*/*b*)^½^) and an unstable one at *x* = 0. For the left stable point to become unstable by merging with the unstable point at the origin, both the first derivative (in eqn. (3.1)) and the second derivative should be zero at the same point.
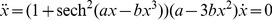
(3.2)or,
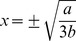
Substituting the last result in eqn. (3.1) (with 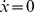), we have
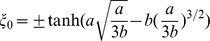
for *a* = *b* = 1, *ξ*_0_ = ±0.366.

Thus the minimum amplitude, *A*_0_, which will produce a transition from resting to target position is 0.366. We chose *A*_0_ = 0.25 for the simulations below, which is insufficient to produce a transition from resting state to target state, i.e., to make a successful reach.

We characterize the effectiveness of the stimulus in terms of the probability of reach, *P*, which is expressed as the ratio of the number of successful attempts at reaching, and the total number of reaching attempts.

Now let us choose a suitable value of stimulus duration, *T* (eqn. (2.2.4)). We seek to use a stimulus duration that is not too long and yet achieves the probability of reach, *P* that is close to 1.

In standard SR systems, typically sinusoidal inputs are presented. The response amplitude then depends on several parameters like stimulus amplitude, *A*_0_, stimulus frequency, *f*, and noise amplitude, *D*. Particularly, the response amplitude component, 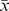, which corresponds to the input frequency, *f*, is given as [29]:
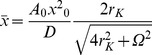
(3.3)where
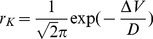
(3.4)is the Kramer rate; Δ*V* is the barrier, the difference between the potential at the minima and the maximum; <*x*_0_^2^> is the *D*-dependent variance of the stationary unperturbed system (*A*_0_ = 0); and *Ω* = 2π*f*. Response amplitude component, 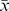, has been shown to reach a maximum for intermediate values of *D*, while the response tends towards a maximum as the stimulus frequency, *f*, tends to 0 [29].

**Figure 4:**
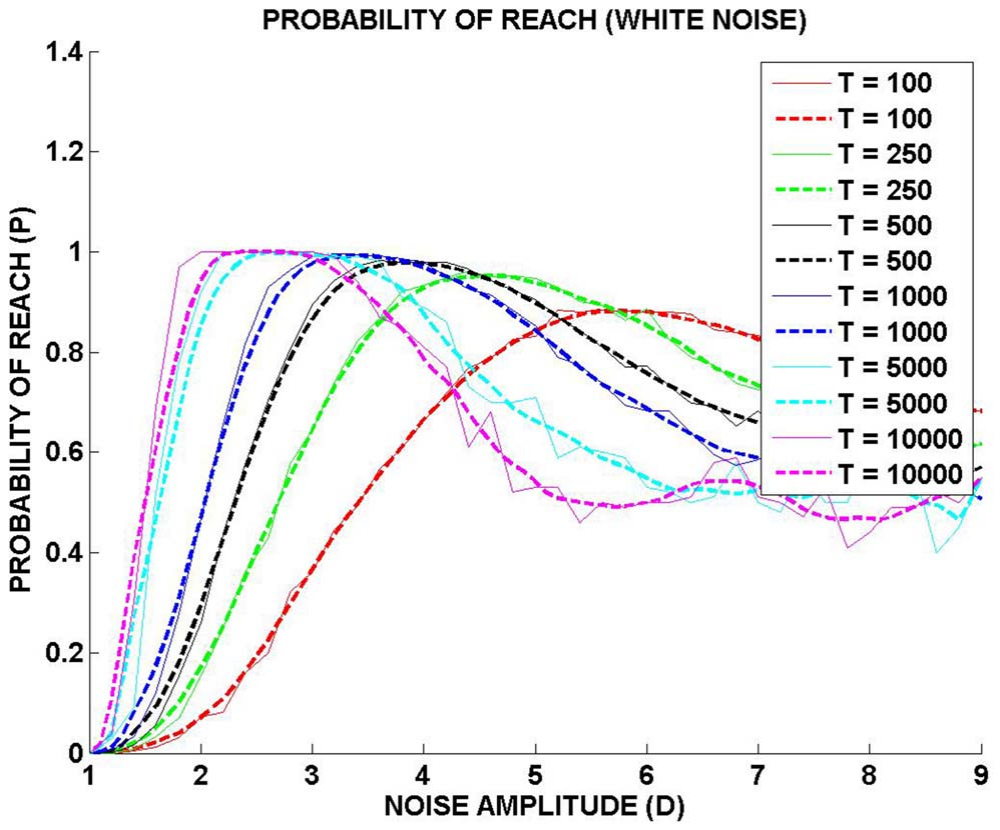
Probability of Reach, *P*, as a function of noise amplitude, *D*, for various values of stimulus duration, *T*. *P* is zero for very low values of *D*: since the stimulus amplitude, *A*_0_, is subthreshold, a minimum level of noise is necessary for a successful reach. Beyond *D* = 4, *P* decreases slowly with increasing *D*. Corresponding to each value of *T*, there is a thin solid line and a thick dashed line. The solid line represents the original simulation result, and the dashed line is the smoother version of the same.

Though the above formula for response magnitude is derived for sinusoidal stimuli, we expect the general trends to be seen in the present case of half-sinusoidal, kick stimulus. We verify this assumption through simulations. Fig. 4 shows the probability of reach, *P*, as a function of noise amplitude, *D*, (stepsize = 0.2) for various values of stimulus duration, *T*. (Probability is calculated by averaging over 1000 trials for each value of *D*). Note that *P* peaks at an intermediate value of *D*, but the peak migrates leftwards as the duration, *T*, is increased. Also note that *P* attains a peak value of 0.996, for *D* = 3.2, in the graph of *P* vs *D* for *T* = 1000 ms (blue graph in fig. 4). Therefore we chose *T = *1000 ms for subsequent simulations. For *T* larger than 1000, the peak of the *P* vs. *T* curve shifts left. In fig. 4, for *T* = 5000 and 10,000, probability of reach equals 1 for smaller values of *D*.

The variability in the *P* vs. *D* plots (fig. 4), particularly for larger noise levels, poses difficulties in finding a unique maximum. Therefore, we smooth the curves before computing the maxima. Smoothing is performed using the following steps:

1. Supersampling: In the original plots, the resolution on x-axis is 0.2. The resolution is doubled by decreasing the stepsize to 0.1 and linearly interpolating the data.
2. Smoothing: Smoothing is performed by simple local averaging over a window size, WIN.

The value of WIN used for fig. 4 is 9. Table 1 shows the maxima of the *P* vs. *T* graphs and the values of *D* at which the maxima occur. Note that *P* values in Table 1 are slightly different from the original data as a result of smoothing process. Though the peak shifts leftwards with increasing *T*, the amount of shift seems to decrease with increasing *T*. It is possible that the peak tends towards a limit as *T* is increased indefinitely.

**Table 1:** 

Stimulus duration *T*	Noise level at peak	Peak Probability of Reaching
100	5.6000	0.8814
250	4.6000	0.9525
500	3.9000	0.9781
1000	3.4000	0.9924
5000	2.6000	0.9983
10000	2.4000	1.0000

But the significance of larger values of *T* from biological point of view must be reconsidered. From a purely mathematical, SR point of view, the best value of *T* is one where highest *P* is obtained with lowest noise. But large values of *T* imply long waiting times before voluntary movements can be initiated, which is not desirable from the perspective of motor efficiency. Therefore, we continue to use *T* = 1000 ( = 1 sec), which is close to the duration of the Readiness Potential, as the baseline result in our simulations. In more realistic, future versions of the model, we will try to use experimental data to choose the right value of *T*.

**Figure 5:**
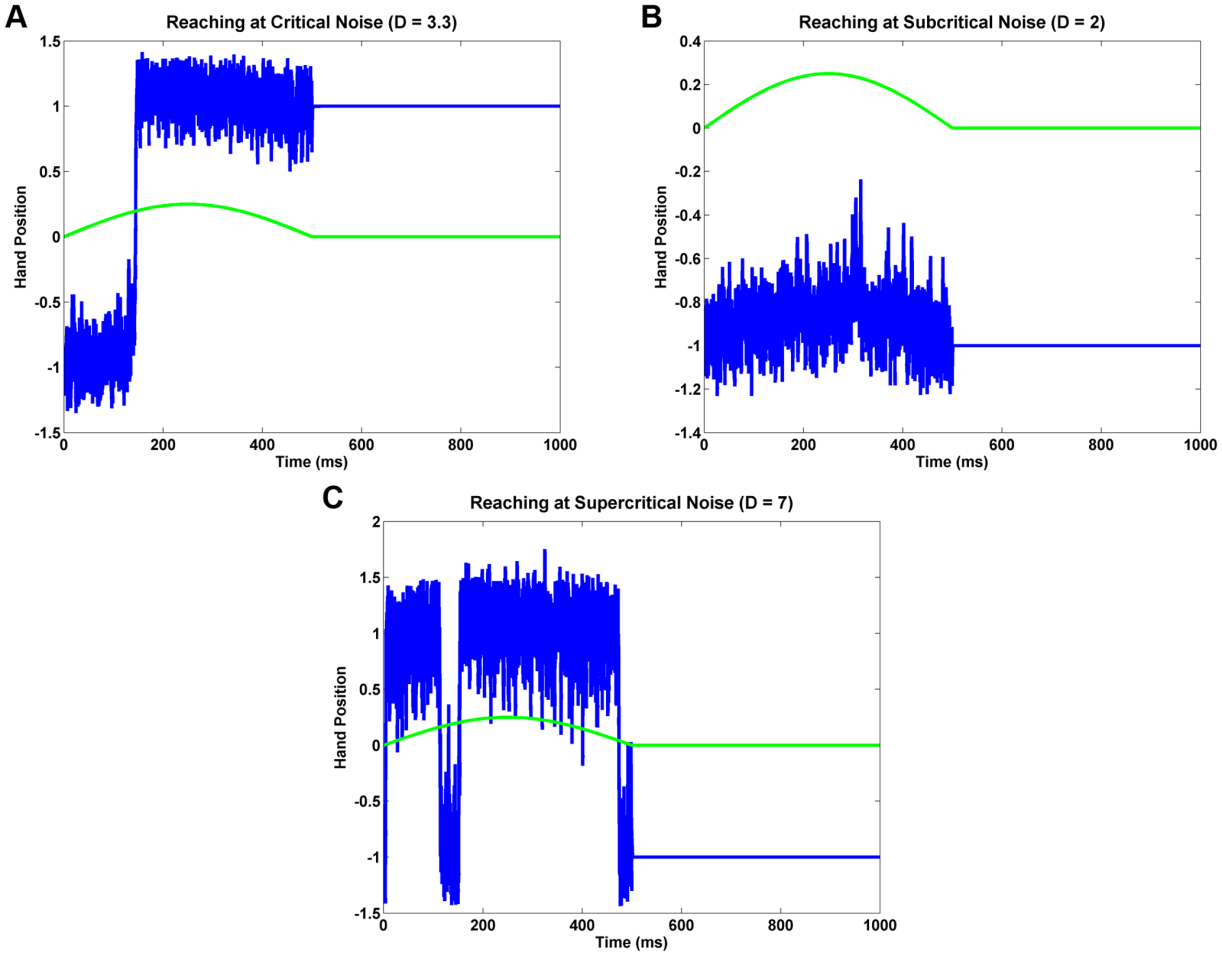
Trajectories of reaching for (a) critical noise amplitude, *D* = 3.3, (b) subcritical noise amplitude, *D* = 2.0, and (c) supercritical noise amplitude, *D* = 7.0. Solid line, which denotes hand position, depicts the transition from the resting position (*x* = −1) to the target position (*x* = 1). Dashed line denotes the stimulus – the sinusoidal “kick” with amplitude *A*_0_ = 0.25.

Figs. 5(a, b, c) show the reaching trajectories for three noise levels: critical (*D* = 3.3), subcritical (*D* = 2) and supercritical (*D* = 7) respectively. We propose that the optimal noise condition (*D* = 3.3), is comparable to the state of BG of a normal individual. We further suggest that under Parkinsonian conditions, noise level changes due to altered dynamics of the IP [9]. Fig. 5b shows an instance of unsuccessful reach due to inadequate noise. Such reaching behavior may be comparable to akinetic rigidity of PD patients. When noise level is higher than the optimum *D* = 6 (fig. 5c), probability of reaching is again reduced due to large fluctuations in hand position. Such behavior is reminiscent of uncontrolled movements of chorea and dyskinesia observed in PD.

The next study is concerned with the effect of colored noise on reaching probability. In SR literature, white noise is replaced with colored noise simply to study the effect of a realistic noise on SR phenomenon [29]. Gammaitoni et al [29] use the following equation to model colored noise:
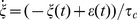
(3.5)where *ε* (*t*) is zero-mean, Gaussian white noise with,<*ε*(*t*) *ε*(0)> = 2*D δ* (*t*), and *ξ*(*t*) is colored noise with, <*ξ*(*t*) *ξ*(0)> = (*D*/*τ*_c_) exp(-|*t*|/*τ*_c_). Increasing correlation time, *τ*_c_, is known to shift the SR peak to the right, implying that it takes stronger noise levels to produce SR with colored noise [29].

The relevance of colored noise in the proposed BG model can be traced to the electrophysiological finding that the activity of STN neurons exhibits increased correlation under dopamine-deficient conditions [36], [37]. The result suggests that under PD conditions the noise arising out of IP may be modeled as colored noise.

**Figure 6:**
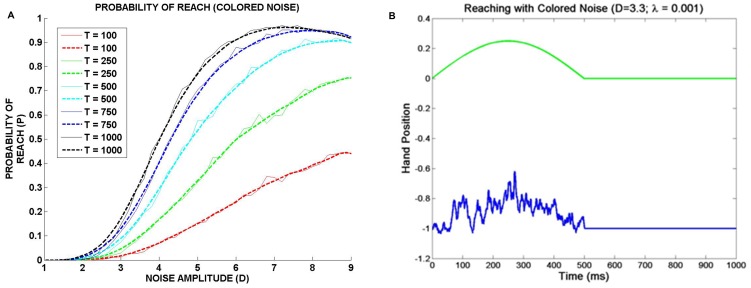
Reaching with colored noise. (a) Plots of probability of reach (*P*) vs. noise amplitude (*D*) for colored noise for various values of *T* ( = 100, 250, 500, 750, 1000). Corresponding to each value of *T*, there is a thin solid line and a thick dashed line. The solid line represents the original simulation result, and the dashed line is the smoother version of the same. *A*_0_ = 0.25. *λ* = 0.001. (b) An instance of reaching under colored noise conditions. *λ* = 0.001, *D* = 3.3, *A*_0_ = 0.25, *T* = 1000.

We simulated colored noise as, 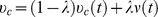, where *λ* = 0.001 and *ν*(*t*) is Gaussian random variable (mean = 0, SD = 1). Fig. 6a shows the probability of reaching as a function of *D*, under colored noise conditions. Note the rightward shift and also reduction in peak probability compared to white noise case of fig. 4. Fig. 6b shows an instance of reaching trajectory for *D* = 3.3 under colored noise condition.

Table 2 shows the maxima of the *P* vs. *T* graphs and the values of *D* at which the maxima occur (WIN = 15). With colored noise also, as it happened with white noise above, the peak shifts leftwards with increasing *T*, the amount of shift seems to decrease with increasing *T*. But note that for a given value of *T*, the colored noise case (fig. 6) requires much larger noise levels to produce the same reaching efficiency, compared to white noise case (fig. 4). Thus it is evident that not just noise amplitude but noise quality also matters in determining reaching efficiency.

**Table 2:** 

Stimulus duration *T*	Noise level at peak	Peak Probability of Reaching
100	8.9	0.4435
250	8.9	0.7530
500	8.8	0.9083
750	7.9	0.9485
1000	7.3	0.9618

Additional simulation results are described in Text S2. Fig. S1 in Text S2 shows the *P* vs *D* results for *T* = 100, 250, 500, 1000, 5000 and 10,000. Maxima of the *P* vs. *T* graphs in fig. S2 in Text S2 and the values of *D* at which the maxima occur are given in Table S1 in Text S2. Fig. S2 in Text S2 shows plots of *P* vs. *D* for colored noise for *T* = 100, 250, 500, 750, 1000. Table S2 in Text S2 shows the maxima of the *P* vs. *T* graphs of fig. S2 in Text S2 and the values of *D* at which the maxima occur in case of colored noise. Fig. S3 in Text S2 shows a plot of fractional time (FT) vs. noise amplitude (*D*). Fractional time refers to the fraction of the time during which the target is reached. This measure is introduced as an alternative to Probability of reach, *P*. Fig. S4 in Text S2 shows a plot of *P* vs. *D* for various values of *ε* in eqn. 2.2.2.

## Discussion

We present an abstract model of the possible role BG play in amplifying willed action. The present model is derived by simplifying an earlier model of the role of BG in reaching movements [24]. The model of [24] is cast in the framework of RL . The outputs of motor cortex and BG are combined to compute the muscle activations necessary to drive the arm towards the target. For a constant output of the motor cortex, the varying BG output actually searches for the appropriate muscle activation vector that can perform a successful reach. This dynamics of the output of BG consists of two components: 1) the dynamics of hill-climbing over a Value function, and 2) a stochastic component corresponding to exploratory behavior in RL. With this combination of hill-climbing, which can be re-interpreted as gradient-ascent over an appropriately defined potential function, and stochastic dynamics, corresponding to the exploratory dynamics of the IP of BG, the proposed BG model has the right ingredients to support SR phenomenon. We propose that BG circuit amplifies weak will signals through such SR effect.

The BG dynamics of eqns. 2.1.6(a, b, c) are rewritten in a form (eqn. 2.2.2) that resembles standard SR dynamics (eqn. 2.2.1) involving gradient descent over a bistable well. The simplified form of eqn. (2.2.2) is used to simulate reaching dynamics in the present study. The two stable states of the potential denote a target position and a resting position respectively. Willed action signal is simulated as a half-sinusoid with a subthreshold amplitude: the signal in itself is insufficient to make a successful reach without added noise. Reaching probability reaches 0.996 at a stimulus duration of *T* = 1000 ms and for *D* = 3.3. For smaller noise levels, reaching probability drops to zero for *D* = 1, and for higher noise levels, reaching probability exhibits a long tail approaching the value of 0.5. Colored noise is simulated as 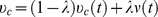 where *λ* = 0.001 and *v*(*t*) is Gaussian random variable (mean 0, SD = 1). Reaching probability profile is shifted to the right in case of colored noise, compared to the case of white noise. Highest reaching probability of 0.96 is achieved for *D* = 7.3.

The present work assumes that the IP in BG is the source of noise necessary for the hypothesized SR dynamics. This assumption has its roots in a line of modeling work that applies RL concepts to understand BG function [21]. There is a growing consensus in contemporary BG research that BG forms a neural substrate for RL [22]. This insight paved way to a large literature of RL-based BG models, most of them addressing only specific aspects of the many functions of BG. Efforts are underway to explain the rich variety of BG functions solely within the RL framework [21].

In RL-based learning, an agent learns to respond to stimuli with actions that maximize future reward. There are three key components in RL framework viz., Actor, Critic and Explorer [21]. The Actor is the module that performs actions, in accordance with a policy that maps states to actions; Critic predicts the total future reward, a quantity known as Value, based on past rewarding experiences; Explorer injects perturbative noise that allows the agent to explore randomly the space of actions. RL-based action selection involves a combination two complementary dynamics: exploitation which consists of climbing up the Value gradient, while exploration refers to stochastic perturbation from greedy gradient ascent over Value profile. It has been hypothesized earlier that the IP is the subcortical substrate for exploration [21], [38].

The IP of BG has been given a variety of interpretations including: withholding of action [9], [23], focusing and sequencing [39], action selection [40], and switching between voluntary and automatic movements [41]. But by assuming that the IP subserves exploration, we find an elegant complementarity between the two pathways whereby the direct pathway (DP) subserves exploitation while the IP supports exploration.

Presence of complex dynamics in the IP lends support to the possibility that IP can have a role in exploration. Degradation of such complex activity to more regular forms of activity like synchronized bursts is hypothesized to contribute to impaired movement. Experimental studies of activity of STN and GPe revealed that under dopamine-depleted circumstances (analogous to Parkinsonian conditions), activity of these nuclei exhibited, though not much reduction in firing rate, a dramatic increase in correlations among neurons [36], [42], [43]. Correlated activity of neurons of STN-GPe loop has been functionally linked to Parkinsonian tremor frequencies [43]. Complex activity of STN-GPe loop in normal BG has been attributed a deep functional significance, and interpreted as a source of stochastic exploratory signal required by RL [38], [21], [44] and degradation of this complex activity due to increased correlations in neural firing has been linked to impaired movement. Experimental evidence that supports the involvement of the IP in exploratory behavior exists. Bilateral lesions of STN is known to induce perseverative behavior, which may be regarded as a form of impaired exploration [45]. High frequency stimulation of STN, which functionally mimics STN lesioning, is also known to induce perseverative behavior [46].

In order to make more precise these intuitions about the possible role of the IP in BG function, we recently developed a neural network model of BG instantiated in an action selection task [34]. In this model, striatal dopamine is assumed to switch between DP and IP activation. The IP is modeled as a loop of the Subthalamic Nucleus (STN) and the Globus Pallidus externa (GPe), capable of producing chaotic activity. Simulations with this model suggest that the classical Go/NoGo picture of BG pathways may have to be expanded. In classical descriptions of BG function, the DP is known as the Go pathway since it facilitates movement and the IP is called the NoGo pathway since it inhibits movement. But simulation results from the model of [34] suggest that while the system displays Go and NoGo regimes for extreme values of dopamine, at intermediate values of dopamine, it exhibits a new Explore regime denoting a random exploration of the space of action alternatives. The exploratory dynamics originates from the chaotic activity of the STN-GPe loop. This chaotic activity of the IP (consisting of STN-GPe loop), which plays the role of an explorer in [24], is represented by the noise source in the present study.

Therefore, the combination of exploitation (gradient ascent over Value function) and exploration (stochasticity) in BG pathways, seem to provide appropriate machinery for SR dynamics. The present work proposes that BG uses this SR dynamics to amplify weak willed action signals. Since the best probability of reaching occurs at an intermediate level of noise (*D*), it can be thought to correspond to normal healthy BG physiology. For lesser noise levels, reaching probability drops, resulting in a situation analogous to hypokinetic symptoms or akinetic rigidity of PD, which is often seen under conditions of OFF medication. The idea receives further support from the fact that overactivation of STN, or GPe lesions cause hypokinetic symptoms [9]. For higher noise levels, reaching probability again drops but for a different reason: the arm exhibits uncontrolled movements and does not stabilize at the target. This is analogous to the situation of overactivation of GPe, or STN lesions, or a state of ON medication, any of which cause hyperkinetic symptoms, or chorea [9]. The case of colored noise may be thought to correspond to increased correlation in STN neural firing patterns under dopamine deficient conditions [36].

**Figure 7:**
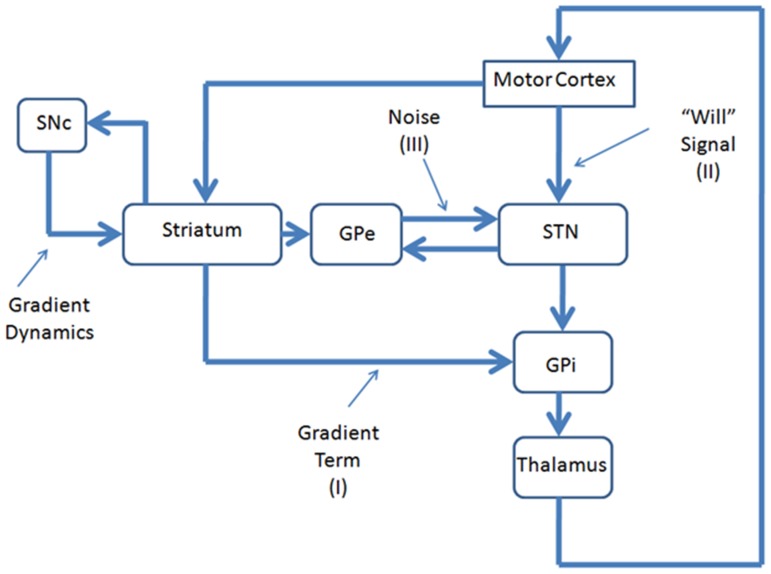
A block -diagram depicting the proposed neurobiological substrates of SR dynamics of eqn. (2.2.2).

A few comments are in order regarding the neurobiological substrates of various terms in SR eqn. (2.2.1). The Value function is computed, as mentioned before, in the striatum. Gradient of value function is computed in the model partly in DP (Go regime, eqn (2.1.6a)) and partly in the IP (NoGo regime, eqn (2.1.6c)). The noise term in eqn. (2.2.1), is thought to arise out of the chaotic dynamics of STN-GPe loop (corresponding to eqn. (2.1.6b)). Identifying the substrate for the willed action signal is more involved. In the introductory section, we presented some early data identifying SMA as perhaps the first brain region that becomes activated before voluntary movement [47]. These early observations have been reconfirmed more recently using a magnetoencephalogram (MEG) with a higher temporal resolution [48]. Studies using fMRI also note activity in both pre-SMA and SMA before activity begins in motor cortex [49]. The link between pre-SMA and willed action crops up in a very different context also. Studies on eye-movement (saccade) generation in primates suggest that the signal from pre-SMA to STN is essential for switching between automatic (involuntary) to volitionally controlled (willed action) saccades [41]. Thus, the signals that correspond to the three terms on the R.H.S. of eqn. (2.2.2) – gradient descent, noise and weak input – come together in the BG circuit. But it still begs the question of the exact site inside BG where the three signals come together and are integrated. A candidate site for such integration is Globus Pallidus interna (GPi), the output nucleus of BG. The aforementioned neural substrates for the proposed BG-based model of willed action are summarized in Table 3 and depicted in fig. 7.

**Table 3:** 

SR component	Neurobiological component
Potential function	Value function computed in striatum
Noise	Chaotic activity of STN-GPe
Forcing signal	Will command from pre-SMA/SMA

The current model of willed action based on SR-like effect subserved by cortico-striatal dynamics is only a preliminary model and must be expanded to more detailed network level models in the future. We must however point out that the present model is derived from a set of RL-based network models of BG developed by our group in the past [1], [21], [34], [44]. The present work only shows that an SR-like effect is buried in the BG dynamics, which can be fruitfully linked to the well-known role of BG in willed action. Though conceptually well placed with respect prior modeling literature on BG, – particularly what belongs to the actor-critic modeling tradition, – the proposed model awaits confirmation by direct experiments on substrates of voluntary movement.

The present model of willed action has some relevance to the study of Ashby et al [30], [50] that discuss the neural substrates for generating automatic movements as opposed to non-automatic movements. Discussing the role of BG in generating automatic movements, Ashby et al [30], [50] suggest that specific parts of the striatum are involved in such movements. During the early stages of motor learning, movements are voluntary and effortful; with practice they become automated. Ashby et al [30], [50] suggest that the associative striatum is active during the early stages of learning, while the activity shifts to sensory-motor striatum when the movements become well-practiced and automatic. Since movements driven by willed action are naturally non-automatic, it is plausible that willed action movements are supported specifically by associative striatum.

There are several instances of amplification of weak sensory stimuli due to SR in animal sensory systems: in mechanosensation of crayfish, cricket and rat [51]–[53]. Similar results were observed in human sensory perception also. Humans were able to detect weak cutaneous stimuli presented to finger, in presence of optimal noise levels [54]. In a study related to auditory perception, humans were asked to discriminate weak, pure tones from white noise signals. Best performance was obtained when optimal noise was added to pure tones [55]. There were several instances of the presence of SR effect in motor function. Cordo et al [56] showed that the sensitivity of muscle spindle receptors can be improved by adding noise to the tendon of the parent muscle. Priplata et al [57] showed that presenting stochastic vibration to insoles improved balance in elderly subjects. Mulavara et al [58] demonstrated that stochastic vestibular stimulation improved ocular stabilization reflex in response to whole-body tilt. Pinamonti et al [59] show stochastic multiresonance in a neural network model and link it to a similar phenomenon observed in human perception [60]. Although there is no direct reference to SR, the work by Todorov [61] on stochastic optimal control highlights the importance of noise in modeling sensory-motor function. The present study appears to be the first modeling attempt to propose a role for SR effect in willed action.

Several neural network models have been constructed to produce SR-like effects without an explicit multistable potential or a simple additive noise term [62]–[65]. For example, Mejias and Longtin [62] presented a heterogeneous spiking neuron network in which the average firing rate of the network is modulated by a weak, periodic input signal. Input/output correlation is found to be the highest at certain optimal heterogeneity parameter revealing an SR-like underlying effect. Neural network models of this kind might give pointers to expansion of the proposed willed action model to its more detailed network versions.

The proposed model shares some features with a recently published model of neural mechanisms underlying self-initiated movement [66]. The model of [66], described as a leaky stochastic accumulator, consists of a gradually accumulating signal with noise added. Movement is released when this accumulating signal crosses a threshold. Due to the presence of noise, the exact time at which the threshold is crossed shows variability. The model is able to accurately explain behavioral and electrophysiological data (waiting times and EEG amplitudes) from human subjects performing self-initiated movements. The accumulation process in the model of [66] is analogous to gradient dynamics, and approach to an attractor, in our model. (An electrophysiology-based study also describes motor preparation in terms of attractor dynamics. Based on recordings from premotor cortex of behaving monkeys, Churchland et al (2006) suggest that neural dynamics underlying motor preparation may be described as approach to an attractor [67]. The noise is analogous to the noise generated by IP in our model. Future efforts will be directed at taking these convergences further and develop a comprehensive neuromotor model of mechanisms underlying willed actions.

### 4.1 Limitations of the Study, Open Questions and Future Work

In this section, we discuss the underlying assumptions, which become limitations, of the proposed work. This preliminary model of the role of BG in willed action shows that a certain form of reinforcement-based learning dynamics of BG, described in our earlier work, has the necessary ingredients for a SR effect. Attributing a meaningful role to this SR effect, we propose that BG's involvement in willed action consists in amplifying a weak will signal by the SR mechanism present in BG machinery.

The proposed model is a lumped model mainly pitched at behavioral level. Therefore the model may appear to be deficient in detailed representation for neurobiological substrates. Since the proposed model is a preliminary model that embodies the seed of an idea, it is kept deliberately simple. But the model can be expanded to more detailed network versions since it has evolved by reduction from detailed network models of BG from our earlier work.

Classical understanding of the functional anatomy of BG describes the DP and IP as the Go and NoGo pathways respectively. Our group has been developing a line of BG models in which DP is still the Go pathway but the IP subserves exploration in addition to the earlier NoGo function. In [21] we outlined how this expanded functional depiction of BG can be used to explain a wide range of BG functions. In [34] we presented a model of BG that exhibits three regimes – Go, NoGo and Explore – with the explore regime emerging out of the chaotic dynamics of the STN-GPe loop. In [44] we present a model of BG involved in saccade generation. BG nuclei involved in saccade generation – Caudate, SNr, STN, GPe are explicitly represented. The model is trained by RL. Value is computed in striatum, dopamine signal corresponds to temporal difference error, and the indirect pathway is the substrate for exploration. A lumped version of the network model described in [44] was used in [1] to model reaching performance in normal and Parkinsonian conditions. Based on the line of work just described it is possible to expand the proposed model of willed action to its network version with appropriate neurobiological elements.

Reaching movement is formulated in the proposed model as a transition from one minimum (resting position) to another (target position) in a bistable potential. Such a scenario of reaching is an oversimplification, since it must be possible to reach a three-dimensional continuum of target positions from a similar continuum of resting positions. To this end, the potential function must be dynamically carved so that the two minima can be placed anywhere within the volume over which the hand is restricted. This can be achieved in a straightforward manner by allowing the resting and target positions parameterize the potential function. These modeling features are ideally incorporated in the expanded network version of the proposed model.

Application of the term SR to the proposed model has to be, strictly speaking, reconsidered since resonance implies an underlying frequency. There has been some debate in the literature about use of the term SR to certain biological phenomena in which noise plays a constructive role [68], [69]. The term resonance is suggestive of frequency resonance which is the case with classical stochastic resonance. But McDonnell and Ward [69] use a more general term known as ‘stochastic facilitation’ which refers to a larger class of phenomena in which noise plays a beneficial role. Phenomena of SR then become a subclass of those of stochastic facilitation. Although the proposed model is presented as a case of SR, it must be noted that the model may not be strictly dubbed as one of classical stochastic resonance. The reason is that in the proposed model the input is not a periodic signal and therefore there is no frequency involved. However, considering the strong resemblance between eqn. (2.2.1) and eqn. (2.2.2) we choose to call the proposed model one of SR. More general future developments of the current model may perhaps be better described as models of stochastic facilitation.

## Conclusions

The proposed model shows that a line of RL-based models of BG has an implicit SR effect. Exploiting the ability of SR effect to amplify a weak signal, we link the SR effect buried in BG dynamics with the functional associations between willed action impairment and BG lesions. Since the proposed model is a lumped model, more detailed network-level models, first with rate-coded neurons, and then with conductance-based neuron models, need to be developed. It will be possible to validate precise predictions that will emerge out of such detailed models using functional imaging techniques.

## Supporting Information

Figure S1 **Plots of ‘probability of reach’ (*P*) vs. noise amplitude (*D*) for white noise for various values of *T*. **Corresponding to each value of *T*, there is a thin solid line and a thick dashed line. The solid line represents the original simulation result, and the dashed line is the smoother version of the same.
(TIF)

Figure S2 **Plots of ‘probability of reach’ (*P*) vs. noise amplitude (*D*) for colored noise for various values of *T*. **Corresponding to each value of *T*, there is a thin solid line and a thick dashed line. The solid line represents the original simulation result, and the dashed line is the smoother version of the same.
(TIF)

Figure S3 **A plot of “fractional time” (FT) vs. noise amplitude (*D*).**
(TIF)

Figure S4 **Plot of ‘probability of reach’ (*P*) vs. noise amplitude (*D*) for various values of ε in eqn. 2.2.2.** For increasing values of ε, the peak of the *P* vs. *D* graph shifts leftwards.
(TIF)

Text S1 **Appendix.**
(DOCX)

Text S2 **Supporting figures and tables.** Figure S1, Plots of ‘probability of reach’ (*P*) vs. noise amplitude (*D*) for white noise for various values of *T*. Corresponding to each value of *T*, there is a thin solid line and a thick dashed line. The solid line represents the original simulation result, and the dashed line is the smoother version of the same. Table S1, Maxima of the *P* vs. *T* graphs in fig. S1 and the values of *D* at which the maxima occur. Figure S2, Plots of ‘probability of reach’ (*P*) vs. noise amplitude (*D*) for colored noise for various values of *T*. Corresponding to each value of *T*, there is a thin solid line and a thick dashed line. The solid line represents the original simulation result, and the dashed line is the smoother version of the same. Table S2, Shows the maxima of the *P* vs. *T* graphs and the values of *D* at which the maxima occur (WIN = 15) for colored noise. Figure S3, A plot of “fractional time” (FT) vs. noise amplitude (*D*). Figure S4, Plot of ‘probability of reach’ (*P*) vs. noise amplitude (*D*) for various values of ε in eqn. 2.2.2. For increasing values of *ε*, the peak of the *P* vs. *D* graph shifts leftwards.
(DOCX)
