# Supplementary figures and images for: Do Basal Ganglia Amplify Willed Action by Stochastic Resonance? A Model
Source: PLoS One. 2013 Nov 26;8(11):e75657. doi: 10.1371/journal.pone.0075657 (PMC3841152; doi:10.1371/journal.pone.0075657)

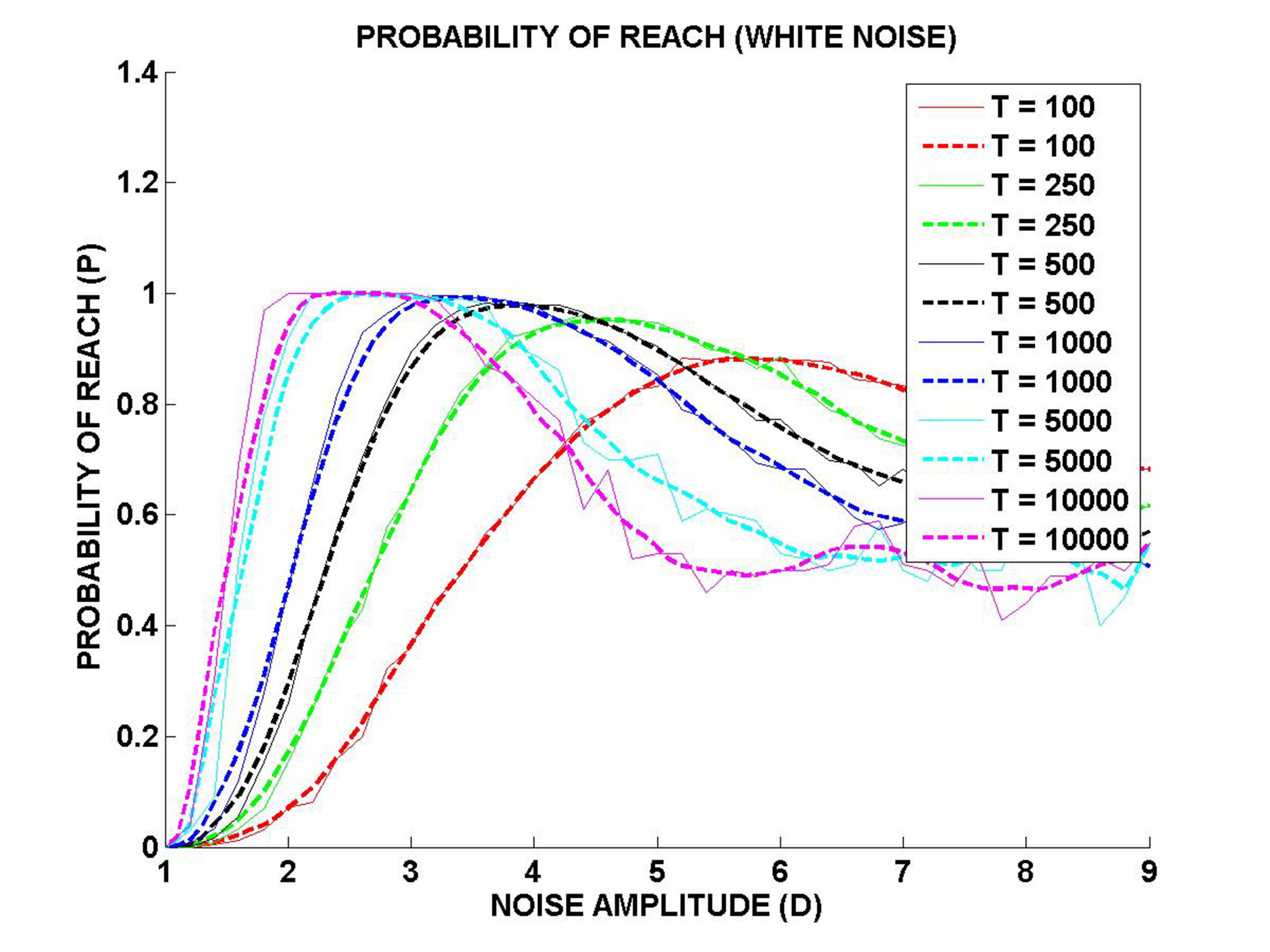

Supplement: Figure S1 — Plots of ‘probability of reach’ (P) vs. noise amplitude (D) for white noise for various values of T. Corresponding to each value of T, there is a thin solid line and a thick dashed line. The solid line represents the original simulation result, and the dashed line is the smoother version of the same. (TIF) [file pone.0075657.s001.tif]

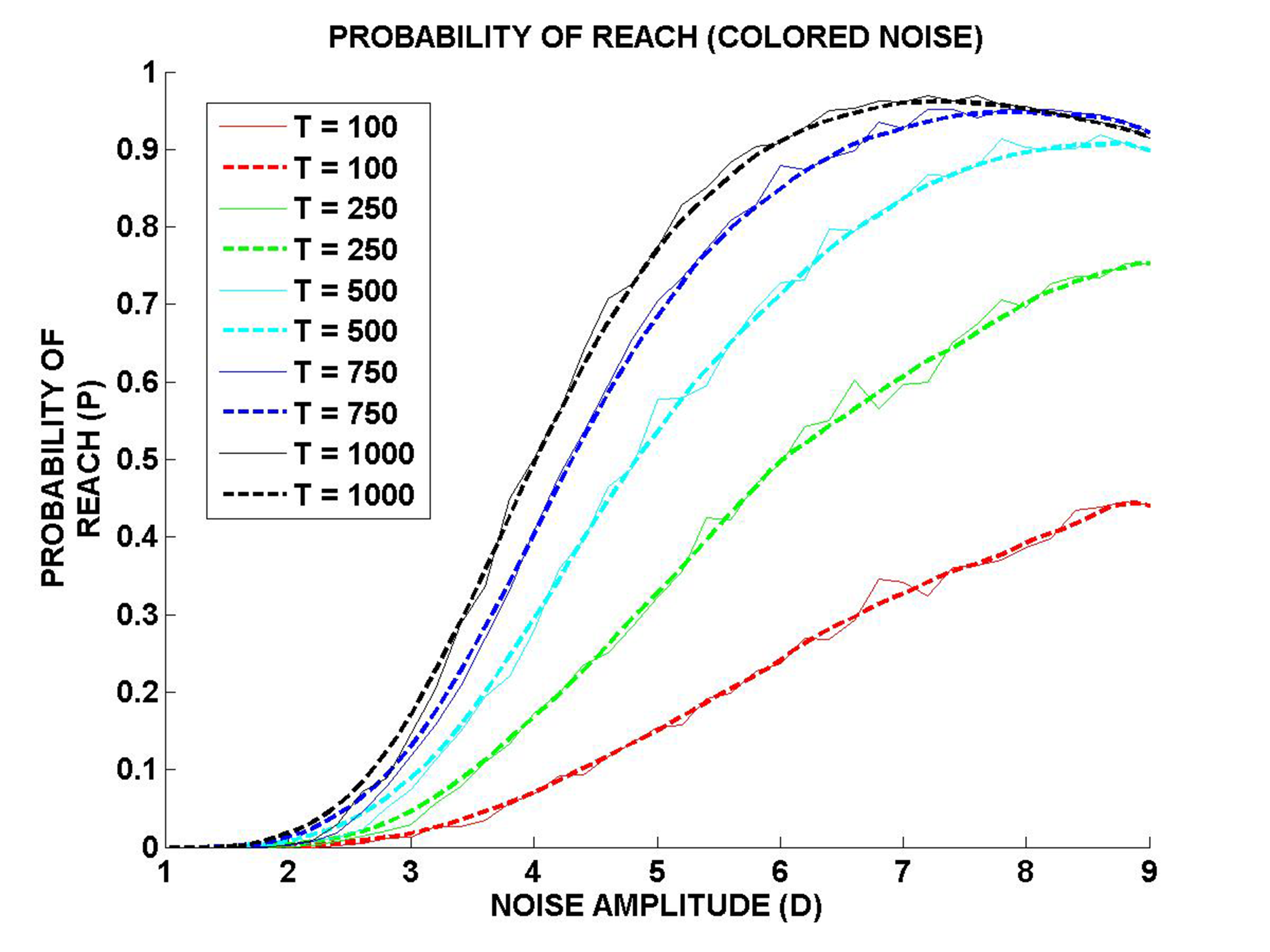

Supplement: Figure S2 — Plots of ‘probability of reach’ (P) vs. noise amplitude (D) for colored noise for various values of T. Corresponding to each value of T, there is a thin solid line and a thick dashed line. The solid line represents the original simulation result, and the dashed line is the smoother version of the same. (TIF) [file pone.0075657.s002.tif]

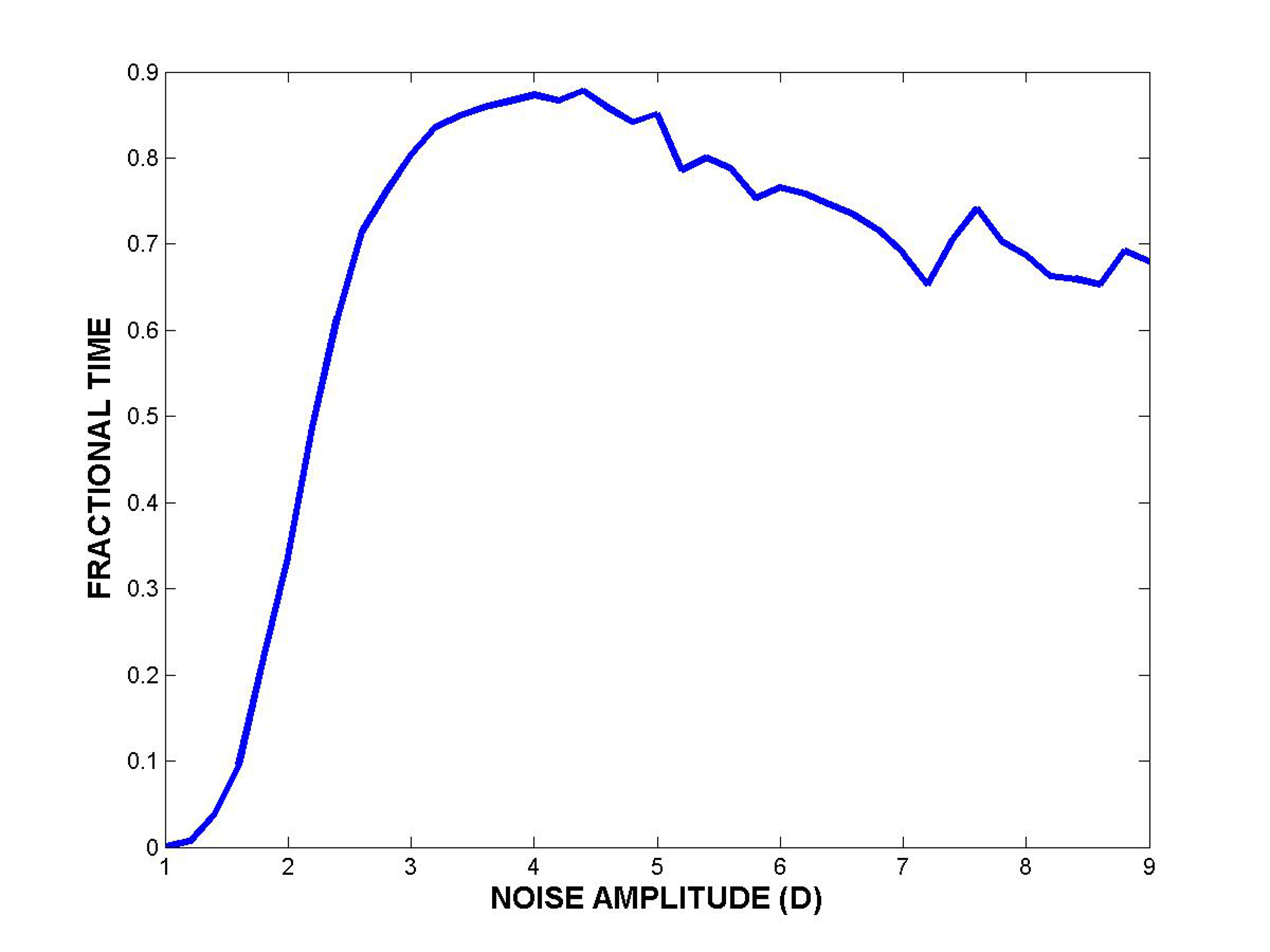

Supplement: Figure S3 — A plot of “fractional time” (FT) vs. noise amplitude (D). (TIF) [file pone.0075657.s003.tif]

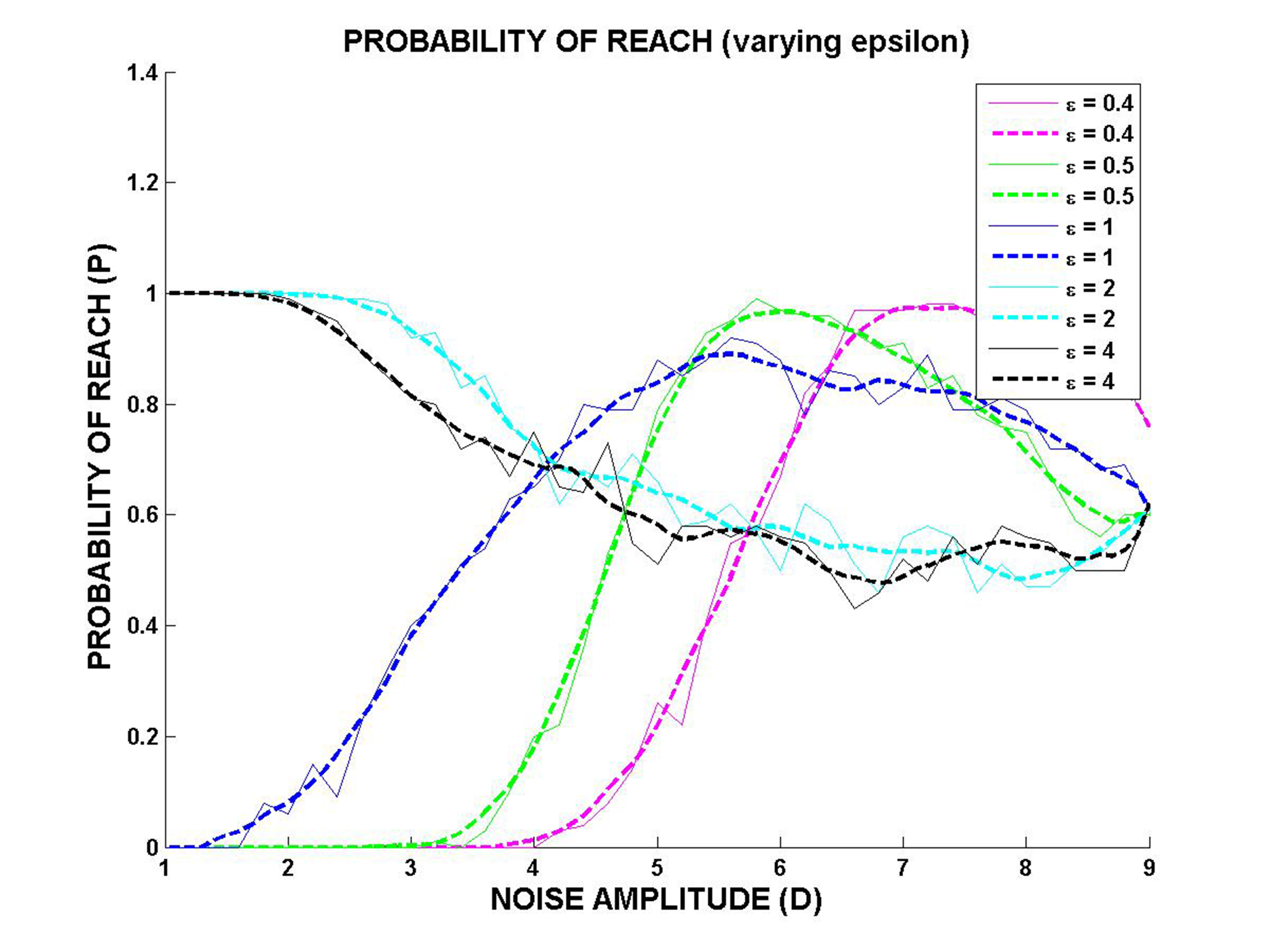

Supplement: Figure S4 — Plot of ‘probability of reach’ (P) vs. noise amplitude (D) for various values of ε in eqn. 2.2.2. For increasing values of ε, the peak of the P vs. D graph shifts leftwards. (TIF) [file pone.0075657.s004.tif]
