# Supplementary material for: Do Basal Ganglia Amplify Willed Action by Stochastic Resonance? A Model
Source: PLoS One. 2013 Nov 26;8(11):e75657. doi: 10.1371/journal.pone.0075657 (PMC3841152; doi:10.1371/journal.pone.0075657)
Supplement: Text S1 — Appendix. (DOCX) [file pone.0075657.s005.docx]

**Appendix**:

The aim is to show that eqns. (2.6,a,b,c) closely resemble SR dynamics of eqn. (3.1). Firstly, let us simplify the notation of eqns. (2.6,a,b,c) as: *g*_bg_ 🡪 *x*, *D*_hi_ = +*ε* Δ*t*, *D*_lo_ = −*ε* Δ*t*. In eqns. (2.6a,b,c), since Δ*V* is an incremental quantity, the thresholds with which it is compared – *D*_hi_ and *D*_lo_ – are also incremental. Note that eqns. (2.6,a,c) denote gradient ascent over the function, *V*, when |Δ*V*| > *ε* Δ*t*. These two equations are combined into a single tanh(.) term (first term on the Right Hand Side (RHS)) of eqn. (A.1) below. Similarly, eqn. (2.6b) denotes a noise term that is significant for |Δ*V*| < *ε* Δ*t*; this is captured by the Gaussian term (second term in eqn. (A.1) below). The third term on the RHS of eqn. (A.1) below represents the willed action term. The fourth term on RHS of eqn. (A.2) represents the stabilizing term. Thus eqns. (2.6a,b,c) can be combined and rewritten, with the new willed action term, as,

$\begin{matrix} \Delta x(t+\Delta t)=C'\frac{\Delta x}{\left|\Delta x\right|}\tanh(\Delta V/(\varepsilon\text{ }\Delta t))+ \\ +A'_{n}\exp(-\Delta V^{2}/(\varepsilon\Delta t)^{2})\phi(t) \\ +A'_{I}\xi(t) \\ +B'\Delta x(t) \end{matrix}$ (A.1)

The term Δ*x*/|Δ*x*| denotes normalization of Δ*x*. Since the Left Hand Side (LHS) of eqn. (A.1) above is incremental, the RHS must also be incremental. We show that all the four terms on RHS of eqn. (A.1) are incremental due, in part, to their dependence on Δ*t*, which is made explicit below.

$\begin{matrix} \Delta x(t+\Delta t)=C\frac{\Delta x}{\left|\Delta x\right|}\tanh(\Delta V/(\varepsilon\Delta t))(\Delta t)^{2}+ \\ +A_{n}\exp(-\Delta V^{2}/(\varepsilon\Delta t)^{2})\phi(t)(\Delta t)^{2} \\ +A_{I}\xi(t)(\Delta t)^{2} \\ +(1-B\Delta t)\Delta x(t) \end{matrix}$ (A.2)

where the coefficients from eqn. (A.1) are redefined in eqn. (A.2) as follows,

*C*’= *C* (Δ*t*)^2^

*A*_n_’= *A*_n_ (Δ*t*)^2^

*A*_I_’= *A*_I_ (Δ*t*)^2^

*B*’= (1- *B* Δ*t*)

$$\begin{matrix} \Delta x(t+\Delta t)-\Delta x(t)+B\Delta x(t)\Delta t=C\frac{\Delta x}{\left|\Delta x\right|}\tanh(\Delta V/(\varepsilon\Delta t))(\Delta t)^{2}+ \\ +A_{n}\exp(-\Delta V^{2}/(\varepsilon\Delta t)^{2})\phi(t)(\Delta t)^{2} \\ +A_{I}\xi(t)(\Delta t)^{2} \end{matrix}$$

Dividing both sides by (Δ*t*)^2^,

$\begin{matrix} \frac{\Delta x(t+\Delta t)-\Delta x(t)}{(\Delta t)^{2}}+B\frac{\Delta x(t)}{\Delta t}=C\frac{\Delta x/\Delta t}{\left|\Delta x/\Delta t\right|}\tanh(\Delta V/(\varepsilon\Delta t)) \\ +A_{n}\exp(-\Delta V^{2}/(\varepsilon\Delta t)^{2})\phi(t) +A_{I}\xi(t) \end{matrix}$ (A.3)

Taking the limit Δ*t* 🡪0, we now convert various terms in eqn. (A.3) above into their respective derivatives. The key term is $\Delta V$/$\Delta t$, which in the limit can be written as,

, where ‘’denotes the gradient operator, and denotes the dot product. The normalization term $|\dot{x}|$ is necessary in the denominator because Δ*V* corresponds to change in *V* due to normalized change in *x* ( = Δ*x*/|Δ*x*|). We thus have,

(A.4)

Some simplifications follow if ‘*x*’ is 1-dimensional.

- Dot product becomes multiplication
- ($\dot{x}/|\dot{x}|$) is the same as sign($\dot{x}$)
- In the first term on RHS of eqn. (A.4) above, ($\dot{x}/|\dot{x}|$) can be absorbed into the tanh()
- In the second term on RHS of eqn. (A.4) above, ($\dot{x}/|\dot{x}|$)^2^ equals 1 almost everywhere.

We thus have,

(A.5)

Assuming sufficiently large *B*, we can ignore the second derivative, and obtain a first order system.

(A.6)

In eqn. (A.6) above, the terms on the RHS are as follows:

- Term I: a gradient-like term
- Term II: noise term
- Term III: Weak signal (willed-action term)

The similarities to the classic SR equation shown below are obvious.

$\dot{x}=-\nabla V_{p}+\psi\left( t \right)+\xi(t)$ (A.7)

Section 3.0 shows that eqn. (A.6) also exhibits SR properties.
