# Supplementary material for: Do Basal Ganglia Amplify Willed Action by Stochastic Resonance? A Model
Source: PLoS One. 2013 Nov 26;8(11):e75657. doi: 10.1371/journal.pone.0075657 (PMC3841152; doi:10.1371/journal.pone.0075657)
Supplement: Text S2 — Supporting figures and tables. Figure S1, Plots of ‘probability of reach’ (P) vs. noise amplitude (D) for white noise for various values of T. Corresponding to each value of T, there is a thin solid line and a thick dashed line. The solid line represents the original simulation result, and the dashed line is the smoother version of the same. Table S1, Maxima of the P vs. T graphs in fig. S1 and the values of D at which the maxima occur. Figure S2, Plots of ‘probability of reach’ (P) vs. noise amplitude (D) for colored noise for various values of T. Corresponding to each value of T, there is a thin solid line and a thick dashed line. The solid line represents the original simulation result, and the dashed line is the smoother version of the same. Table S2, Shows the maxima of the P vs. T graphs and the values of D at which the maxima occur (WIN = 15) for colored noise. Figure S3, A plot of “fractional time” (FT) vs. noise amplitude (D). Figure S4, Plot of ‘probability of reach’ (P) vs. noise amplitude (D) for various values of ε in eqn. 2.2.2. For increasing values of ε, the peak of the P vs. D graph shifts leftwards. (DOCX) [file pone.0075657.s006.docx]

1. In fig. S1, which depicts the relation between probability of reach (*P*) and noise amplitude (*D*), it may be noted that the peak of the *P* vs *D* curve shifts to the left with increasing *T*.

Fig. S1 shows the *P* vs *D* results for *T* = 100, 250, 500, 1000, 5000 and 10,000. The peak indeed continues to shift for *T* = 5000 and 10,000, but the amount of shift is marginal with increasing *T*. For *T* = 10,000, it may be noted that *P* attains 1 for *D* between 2 and 3, whereas for *T* = 5000, *P* equals 1 for *D* between 2.2 and 2.8. Larger values of *T* were not explored due to practical constraints.


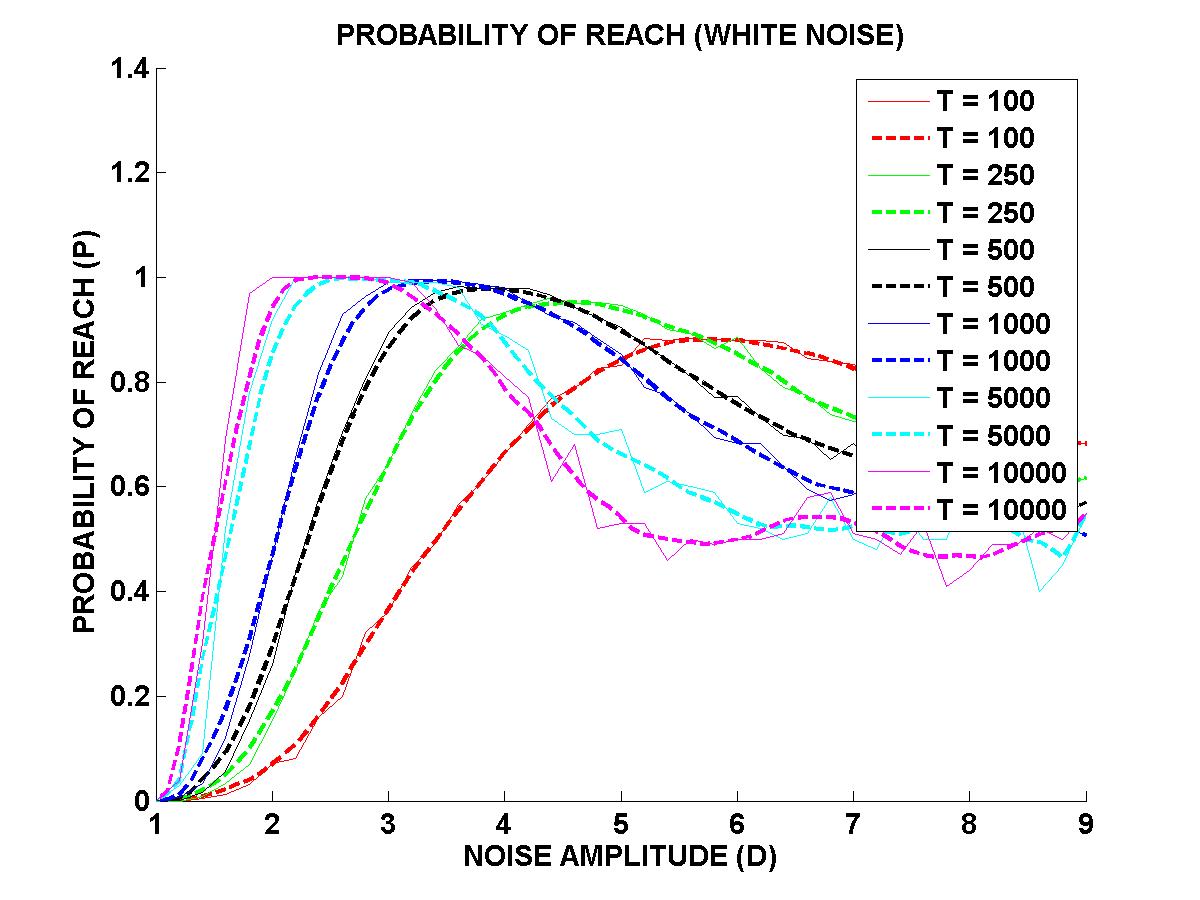


Figure S1: Plots of ‘probability of reach’ (*P*) vs. noise amplitude (*D*) for white noise for various values of *T*. Corresponding to each value of *T*, there is a thin solid line and a thick dashed line. The solid line represents the original simulation result, and the dashed line is the smoother version of the same.

The variability in the Probability of reach, *P* vs. *D* plots, particularly for larger noise levels, poses difficulties in finding a unique maximum. Therefore, we smooth the curves before computing the maxima. Smoothing is performed using the following steps:

1. Supersampling: In the original plots, the resolution on x-axis is 0.2. The resolution is doubled by decreasing the stepsize to 0.1 and linearly interpolating the data.

2. Smoothing: Smoothing is performed by simple local averaging over a window size, WIN.

The value of WIN used for fig. S1 is 9. Table S1 shows the maxima of the *P* vs. *T* graphs and the values of *D* at which the maxima occur. Note that *P* values in Table S1 are slightly different from the original data as a result of smoothing process. Though the peak shifts leftwards with increasing *T*, the amount of shift seems to decrease with increasing *T*. It is possible that the peak tends towards a limit as *T* is increased indefinitely.

| Stimulus duration *T* | Noise level at peak | Peak Probability of Reaching |
| --- | --- | --- |
| 100 | 5.6000 | 0.8814 |
| 250 | 4.6000 | 0.9525 |
| 500 | 3.9000 | 0.9781 |
| 1000 | 3.4000 | 0.9924 |
| 5000 | 2.6000 | 0.9983 |
| 10000 | 2.4000 | 1.0000 |

Table S1: Maxima of the *P* vs. *T* graphs in fig. S1 and the values of *D* at which the maxima occur.

But the significance of larger values of *T* from biological point of view must be reconsidered. From a purely mathematical, SR point of view, the “best” value of *T* is one where highest *P* is obtained with lowest noise. But large values of *T* imply long waiting times before voluntary movements can be initiated, which is not desirable from the perspective of motor efficiency. Therefore, we continue to use *T* = 1000 ( = 1 sec), which is close to the duration of the Readiness Potential, as the baseline result in our simulations. In more realistic, future versions of the model, we will try to use experimental data to choose the right value of *T*.

(2) Considering the fluctuation in the *P* vs *D* graph, it is desirable to perform simulations for larger number of trials, say, for 10,000 trials, instead of 100 trials.

The simulations are running into days even with 1000 trials, on the best workstation accessible to the author. Therefore it was not feasible to run simulations for 10,000 trials. Fig. 4 shows *P* vs. *D* graphs averaged over 1000 trials for *T* = 100, 250, 500, 1000, and for 100 trials for *T* = 5000 and 10,000.

(3) In fig. S2 colored noise simulations are shown for *T* = 100, 250, 500, 750, 1000.


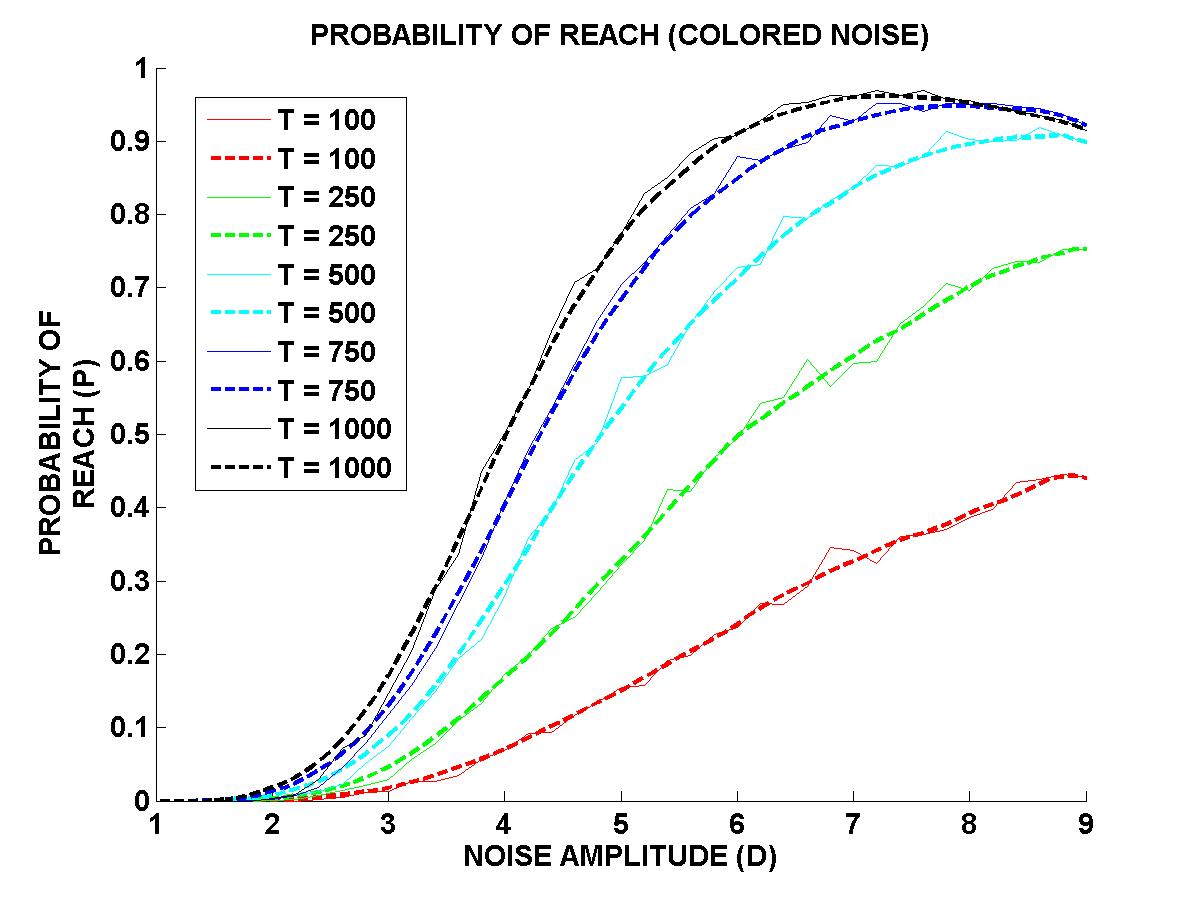


Figure S2: Plots of ‘probability of reach’ (*P*) vs. noise amplitude (*D*) for colored noise for various values of *T*. Corresponding to each value of *T*, there is a thin solid line and a thick dashed line. The solid line represents the original simulation result, and the dashed line is the smoother version of the same.

Table S2 shows the maxima of the *P* vs. *T* graphs and the values of *D* at which the maxima occur (WIN = 15) for colored noise. With colored noise also, as it happened with white noise above, the peak shifts leftwards with increasing *T*, the amount of shift seems to decrease with increasing *T*. But note that for a given value of *T*, the colored noise case (fig. S2) requires much larger noise levels to produce the same reaching efficiency, compared to white noise case (fig. S1). Thus it is evident that not just noise amplitude but noise quality also matters in determining reaching efficiency.

| Stimulus duration *T* | Noise level at peak | Peak Probability of Reaching |
| --- | --- | --- |
| 100 | 8.9 | 0.4435 |
| 250 | 8.9 | 0.7530 |
| 500 | 8.8 | 0.9083 |
| 750 | 7.9 | 0.9485 |
| 1000 | 7.3 | 0.9618 |

Table S2: Shows the maxima of the *P* vs. *T* graphs and the values of *D* at which the maxima occur (WIN = 15) for colored noise.

(4) Since the probability of reaching, *P*, denotes only the final value of position, *x*, in order to get an idea of average behavior of *x*, one may consider an additional measure that quantifies the fraction of time when the target is reached, which can be used instead of *P*. Fig. S3 below shows this Fractional Time (FT). The fractional time curve is quite flat for larger values of *D*. The measure reveals the asymmetry in the relation between reaching effectiveness and *D*. But it may be argued that *P* is a better measure of reaching effectiveness than FT since it denotes where the hand settles at the end of stimulation.


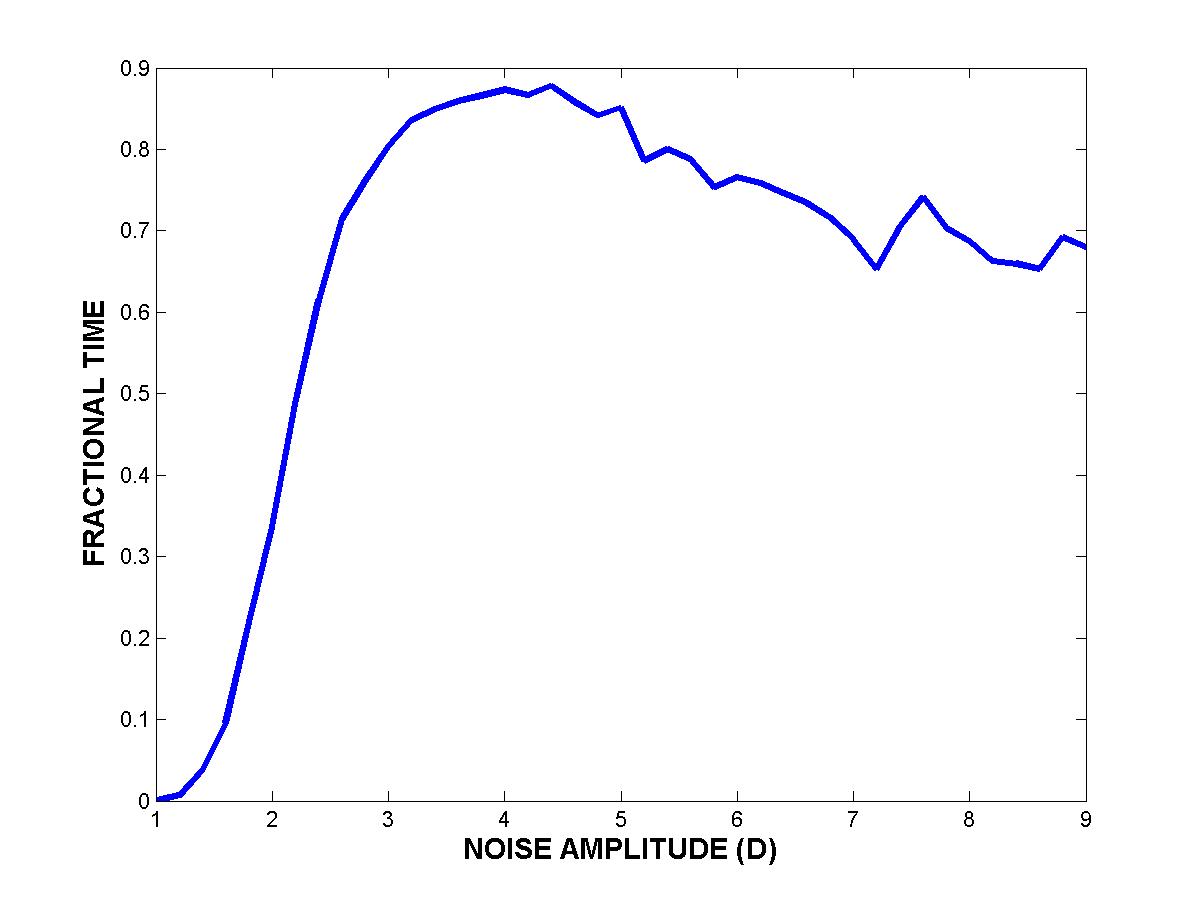


Figure S3: A plot of “fractional time” (FT) vs. noise amplitude (*D*).

5) A question may be asked regarding the choice of the condition *T* = 1000 over the case of *T* = 250, since the *P* vs. *D* curve for *T* = 250 has a longer tail. The answer to the question is as follows:

When trained for 1000 trials, the *P* vs *D* curve for *T* = 1000 has a clear peak at *D* = 3.2, at which point *P* = 0.996 and not 1. *T* = 250 curve indeed has a slower decay beyond the peak, but one does not see an advantage in it. Furthermore, at the peak of the *T* = 250 curve, *P* = 0.96 only and does not approach 1.

Another reason behind choice of *T* = 1000 is given in the manuscript on page 18, and reproduced below:

“But the significance of larger values of *T* from biological point of view must be reconsidered. From a purely mathematical, SR point of view, the best value of *T* is one where highest *P* is obtained with lowest noise. But large values of *T* imply long waiting times before voluntary movements can be initiated, which is not desirable from the perspective of motor efficiency. Therefore, we continue to use *T* = 1000 (= 1 sec), which is close to the duration of the Readiness Potential, as the baseline result in our simulations.”

6) Color noise simulations are now repeated for *T* = 100, 250, 500, 750 and 1000 (fig. S2).

Optimal range of values could be proposed for *D* in the colored noise condition. Then optimal range of values for *D* for both noise conditions could be discussed, as it seems that they are quite different.

7) Explore the impact on performance of the exploratory phase. For that, the value of thresholds *D*_lo_ and *D*_hi_ could be modified. One could, for instance, define a "distance" such as *d* = *D*_hi_ - *D*_lo_ and use it as a parameter to show the variation in performance.

The parameters *D*_hi_ and *D*_lo_ are present in eqn. 2.1.6. The quantity analogous to *D*_hi_ - *D*_lo_ in eqn. 2.2.2 is *ε*. Therefore we vary *ε* and evaluate performance. (fig. S4)


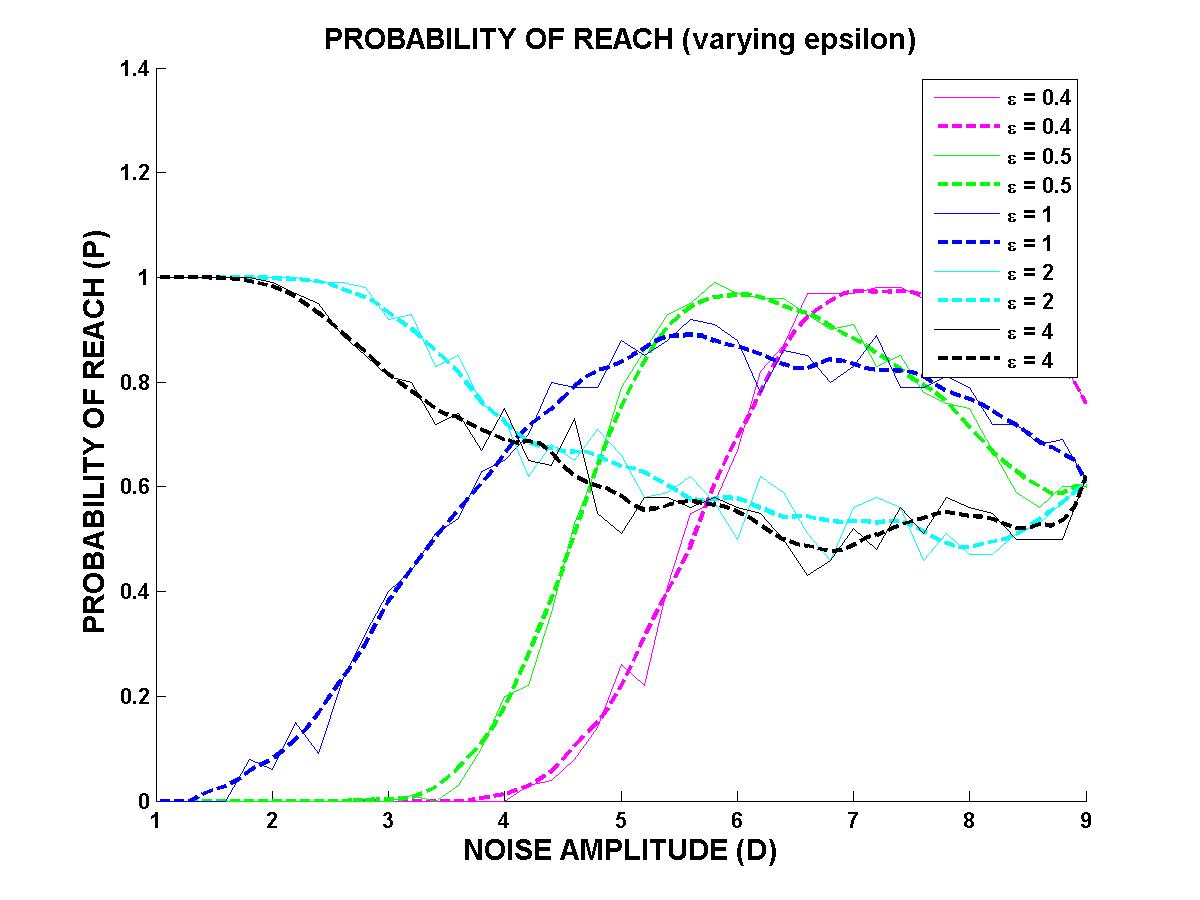


Figure S4: Plot of ‘probability of reach’ (*P*) vs. noise amplitude (*D*) for various values of ε in eqn. 2.2.2. For increasing values of *ε*, the peak of the *P* vs. *D* graph shifts leftwards.
